# Could the Long-Term Oncological Safety of Laparoscopic Surgery in Low-Risk Endometrial Cancer also Be Valid for the High–Intermediate- and High-Risk Patients? A Multi-Center Turkish Gynecologic Oncology Group Study Conducted with 2745 Endometrial Cancer Cases. (TRSGO-End-001)

**DOI:** 10.3390/curroncol28060368

**Published:** 2021-10-29

**Authors:** Mehmet Ali Vardar, Ahmet Baris Guzel, Salih Taskin, Mete Gungor, Nejat Ozgul, Coskun Salman, Umran Kucukgoz-Gulec, Ghanim Khatib, Cagatay Taskiran, Ilkkan Dünder, Firat Ortac, Kunter Yuce, Cosan Terek, Tayup Simsek, Aydın Ozsaran, Anil Onan, Gonca Coban, Samet Topuz, Fuat Demirkiran, Ozguc Takmaz, M. Faruk Kose, Ahmet Gocmen, Gulsah Seydaoglu, Derya Gumurdulu, Ali Ayhan

**Affiliations:** 1 Gynecologic Oncology Department, Medical Faculty, Çukurova University, Adana 01380, Turkey; mavardar@gmail.com (M.A.V.); abguzel@gmail.com (A.B.G.); ukucukgoz@yahoo.com (U.K.-G.); 2 Gynecologic Oncology Department, Medical Faculty, Ankara University, Ankara 06100, Turkey; salihtaskin@yahoo.com (S.T.); mtgungor@gmail.com (M.G.); dunderilkkan@gmail.com (I.D.); fortac@gmail.com (F.O.); 3 Gynecologic Oncology Department, Medical Faculty, Acıbadem University, İstanbul 34684, Turkey; ozguctakmaz@gmail.com; 4 Gynecologic Oncology Department, Medical Faculty, Hacettepe University, Ankara 06230, Turkey; nozgul@gmail.com (N.O.); csalman@hacettepe.edu.tr (C.S.); kyuce@hacettepe.edu.tr (K.Y.); 5 Gynecologic Oncology Department, Medical Faculty, Gazi University, Ankara 06560, Turkey; cagataytaskiran@yahoo.com (C.T.); maonan@gazi.edu.tr (A.O.); 6 Gynecologic Oncology Department, Medical Faculty, Koç University, İstanbul 34450, Turkey; 7 Gynecologic Oncology Department, Medical Faculty, Ege University, İzmir 35040, Turkey; terekmc@yahoo.com (C.T.); ozsarana@hotmail.com (A.O.); 8 Gynecologic Oncology Department, Medical Faculty, Akdeniz University, Antalya 07070, Turkey; tayupsimsek@gmail.com; 9 Gynecologic Oncology Department, Adana Training and Research Hospital, Başkent University, Adana 01250, Turkey; drgoncacoban@yahoo.com; 10 Gynecologic Oncology Department, Çapa Medical Faculty, İstanbul University, İstanbul 34093, Turkey; samettopuz@yahoo.com; 11 Gynecologic Oncology Department, Medical Faculty, İstanbul University, Cerrahpaşa, İstanbul 34098, Turkey; demirkiranfuat@gmail.com; 12 Gynecologic Oncology Department, Medical Faculty, Medipol University, İstanbul 34200, Turkey; mfarukkose@outlook.com; 13 Gynecologic Oncology Department, Umraniye Training and Research Hospital, İstanbul 34760, Turkey; gocmenahmet@yahoo.com; 14 Department of Biostatistics, Medical Faculty, Çukurova University, Adana 01380, Turkey; gulsahseydaoglu@gmail.com; 15 Department of Gynecologic Pathology, Medical Faculty, Çukurova University, Adana 01380, Turkey; gumurdulu@yahoo.com; 16 Gynecologic Oncology Department, Medical Faculty, Başkent University, Ankara 06490, Turkey; aliayhan@baskent.edu.tr

**Keywords:** high-risk endometrial cancer, laparoscopic surgery, survival

## Abstract

This study was conducted to compare the long-term oncological outcomes of laparotomy and laparoscopic surgeries in endometrial cancer under the light of the 2016 ESMO-ESGO-ESTRO risk classification system, with particular focus on the high–intermediate- and high-risk categories. Using multicentric databases between January 2005 and January 2016, disease-free and overall survivals of 2745 endometrial cancer cases were compared according to the surgery route (laparotomy vs. laparoscopy). The high–intermediate- and high-risk patients were defined with respect to the 2016 ESMO-ESGO-ESTRO risk classification system, and they were analyzed with respect to differences in survival rates. Of the 2745 patients, 1743 (63.5%) were operated by laparotomy, and the remaining were operated with laparoscopy. The total numbers of high–intermediate- and high-risk endometrial cancer cases were 734 (45%) patients in the laparotomy group and 307 (30.7%) patients in the laparoscopy group. Disease-free and overall survivals were not statistically different when compared between laparoscopy and laparotomy groups in terms of low-, intermediate-, high–intermediate- and high-risk endometrial cancer. In conclusion, regardless of the endometrial cancer risk category, long-term oncological outcomes of the laparoscopic approach were found to be comparable to those treated with laparotomy. Our results are encouraging to consider laparoscopic surgery for high–intermediate- and high-risk endometrial cancer cases.

## 1. Introduction

Endometrial cancer is the most common gynecological cancer among women in the developed world [1]. Surgery, including total hysterectomy (TH), bilateral salpingo-oophorectomy (BSO) with or without lymphadenectomy and omentectomy is the mainstay of endometrial cancer treatment [1,2,3]. Postoperative adjuvant treatments are decided according to the risk category of the patients. Recently, the joint consensus committee of the European Society for Medical Oncology, the European Society of Gynaecological Oncology and the European Society of Gynaecological Oncology (ESMO-ESGO-ESTRO) classified endometrial cancer as low-, intermediate-, high–intermediate- and high-risk categories in order to tailor the adjuvant therapy after surgery [4]. Surgery for endometrial cancer can be performed either by laparotomy (LT) or laparoscopy (LS). In the 1990s, laparoscopic surgery on gynecologic malignancies was pioneered by the studies of Dargent et al. [5] and Querleu et al. [6] from France and Childers et al. [7] and Spirtos et al. [8] from the United States [9]. Nevertheless, since the publication of the LACC study, in which the laparoscopic approach was found to be associated with poor oncological outcomes in cervical cancer, concerns regarding laparoscopy in gynecological malignancies have been raised [10]. On the other side, the safety of laparoscopy in endometrial cancer has been previously demonstrated in the randomized GOG lap2 and LACE studies [11,12]. Numerous studies, including the abovementioned landmark studies, have found comparable safety and oncological outcomes between laparotomy and laparoscopic modalities in low-risk endometrial cancer [3,11,12,13,14,15,16,17]. However, such studies on high-risk patients are scarce [18,19,20,21,22]. Therefore, it is necessary to increase the studies investigating the oncological safety of laparoscopic surgery in endometrial cancer, particularly those encompassing the high-risk category. Hence, as the Turkish Society of Gynecologic Oncology (TRSGO), we designed this multi-centric retrospective study to compare the oncologic outcomes of laparotomy and laparoscopic surgeries in endometrial cancer, focusing on the high–intermediate- and high-risk categories.

## 2. Materials and Methods

Data were collected from 12 TRSGO centers between January 2005 and January 2016, and they were entered by the investigators of each center and controlled by the same biostatistician (GS). Approval for this study was obtained from the Research Ethics Committee at Çukurova University Faculty of Medicine (10 April 2020, No: 98–15). Written informed consent for the use of research and educational purpose was attained from all patients. All the participating centers are well-known for their long-standing experience in laparoscopic surgery for endometrial cancer. Expert gynecologic pathologists in each of these centers evaluated the pathological materials.

A data sheet of variables related to demographic, clinical, surgical, pathological, follow-up and survival characteristics was recorded from the databases of each center. Only cases with comprehensive data were included, and finally, 2745 endometrial cancer patients were selected for analysis. Age, body mass index (BMI), parity, comorbidities and surgical variables including type of surgery, conversion rate of laparoscopy to laparotomy, duration of surgery, fall in hemoglobin level, postoperative stay in the hospital (day), intraoperative and postoperative complications, stage, grade, histopathological type, myometrial invasion (MI: as <50% and ≥50%), lymph node (LN) involvement, lymphovascular space invasion (LVSI) and number of harvested lymph nodes were recorded. Adjuvant therapy modalities, follow-up data and survival outcomes were also gathered.

All patients’ diagnoses were made upon a preoperative endometrial biopsy. All patients were preoperatively assessed with transvaginal ultrasonography and chest x-ray. Based on the clinician’s decision, further preoperative screenings such as abdomen and thorax computed tomography or magnetic resonance imaging were administered, particularly when there was a suspicion of extra-uterine disease or >50% MI, and in cases of grade 3 or type 2 carcinomas. As this was a retrospective study, no standard preoperative selection criteria were taken into account for laparoscopic surgery. Unless in case of extrauterine disease existence on the imaging methods, LT was preferred. Similarly, no standardization was set among the institutions in this study regarding patients’ discharge decisions. Staging was adapted according to the International Federation of Gynecology and Obstetrics’ 2009 surgical staging classification. All cases underwent TH and BSO, either in LT arm or in LS arm. Uterine manipulator was used for all laparoscopically operated cases. Intraoperative frozen section was applied for all included cases, and a decision to pursue or not to pursue lymphadenectomy was taken based on its result. Lymphadenectomy was not performed in patients with stage 1a, grade 1–2, <2 cm tumors (low-risk factors). Pelvic ± para-aortic lymphadenectomy (± omentectomy) was considered compatible with the Mayo clinic protocol n the presence of any of the following circumstances: endometrioid adenocarcinoma grade 3, tumor diameter >2 cm, ≥50% MI, stage >1a or non-endometrioid histologies [23]. Adjuvant therapies (brachytherapy, external beam radiotherapy and/or chemotherapy) were kept in view for patients with ≥ intermediate risk factors.

A sub-analysis of survival was made between laparoscopy and laparotomy groups according to the pathologic prognostic factors including stage, grade, MI, LVSI, LN status, histopathological type and the recent ESMO-ESGO-ESTRO risk classification system for endometrial cancer [4]. In the ESMO-ESGO-ESTRO classification, patients with Stage I endometrioid, grade 1–2, <50% myometrial invasion and LVSI negative were described as low risk and patients with stage 1 endometrioid, grade 1–2, ≥50% MI and LVSI negative were defined as intermediate risk. High–intermediate risk category was identified as patients with stage I endometrioid, grade 3 and <50% MI, regardless of LVSI status or with stage I endometrioid, grade 1–2 and LVSI unequivocally positive, regardless of MI. High-risk category was decided to include patients with: 1–Stage I endometrioid, grade 3, ≥50% MI, regardless of LVSI status; 2–Stage II endometrioid; 3–Stage III endometrioid, no residual disease; and 4–Non-endometrioid histologies (serous, clear-cell, undifferentiated carcinoma, carcinosarcoma).

Data were analyzed using the SPSS software, version 20.0 (IBM, Armonk, NY, USA). The variables were shown as mean ± standard deviation, median (minimum–maximum) or *n* (%). The variables were analyzed firstly to detect whether or not they were normally distributed by visual (histograms, probability plots) and analytical methods (Shapiro–Wilk’s test). Independent T-test and Chi-square test were used for comparisons between the groups. The follow-up period was accepted as the time between patient’s primary surgery and the last contact. Disease-free survival was designated as the duration (months) from surgery to recurrence. Overall survival was described as the period in months between the diagnosis date and death or last follow-up. The effect of clinico-pathological variables, ESMO-ESGO-ESTRO risk groups and type of surgery on disease-free and overall survival rates of the patients were analyzed using Kaplan–Meier method. Log-rank test was used to calculate the differences among the survival curves. The significance of multiple variables was assessed using the Cox proportional hazard model without violating the proportional hazards assumption.

## 3. Results

Overall, 2745 patients with endometrial cancer were enrolled in this study. Of them, 1743 (63.5%) cases were operated by LT, and 1002 (36.5%) were operated with conventional LS. Demographic and surgical characteristics of the groups are summarized in Table 1. The mean ages of the laparotomy and laparoscopy groups were 59.3 ± 10.7 and 57.5 ± 10.1, respectively. Patients <50 years were 16.5% and 18.8% in the LT and LS groups, respectively. The mean BMI was significantly higher in the LS arm (38.8 ± 4.79) than in the LT (32.1 ± 5.9). Only TH + BSO was performed for 510 (29%) patients in the LT and 329 (32.9%) patients in the LS groups. Staging procedures (retroperitoneal lymphadenectomy ± omentectomy) were added to TH-BSO in the remaining cases of both groups. The mean resected LN number in patients who underwent pelvic lymphadenectomy was 16.3 in the LT vs. 20.9 in the LS groups (*p* < 0.001) and 35.5 in the LT vs. 40.9 in the LS groups for patients who underwent pelvic plus para-aortic lymphadenectomy (*p* < 0.001). Perioperative and postoperative complications were significantly lower in the LS group compared to the LT group (3% vs. 4.8%; *p* < 0.001 and 4.3% vs. 7.6%; *p* < 0.001, respectively). However, peri- and postoperative complications related to urinary and intestinal injuries were noticed more frequently in the LS group. The operation time was significantly lower in the LT group, while the estimated blood loss, drop in hemoglobin and postoperative hospital stay were significantly lower in the LS group. Conversion from LS to LT was required in 27 (3%) cases.

The histopathological results of the LT and LS groups are documented in Table 2. Stage 3–4 cases were noted in 23.9% of the LT arm vs. 11.3% of the LS arm. Grade 3 cases were determined as 12.7% of the LT group and 7.8% of the LS group. The ratios of grade 3 and advanced stage were significantly higher in the LT group than in the LS group. Type 2 (non-endometrioid) histologies were reported in 20.9% and 12.1% of the LT and LS groups, respectively (*p* < 0.001). More than 50% MI was observed in 38.2% and 25.3% of LT and LS groups, respectively (*p* < 0.001). The cervix was invaded in 16.5% and 4.6% of the LT and LS arms, respectively (*p* < 0.001). LVSI was found in 36% of the LT and 22.7% of the LS arm (*p* < 0.001). The removed lymph nodes count was 1–20, 21–40, >40 in 26.3%, 26.1%, 18.3% and 26.7%, 25.9%, 14.6% of the LT and LS groups, respectively. The metastatic LN ratio was 13.2% in the LT group vs. 7.1% in the LS group (*p* < 0.001). Isolated para-aortic LN metastases were recorded in 2.3% and 0.7% of LT and LS cases, respectively.

**Figure 1:**
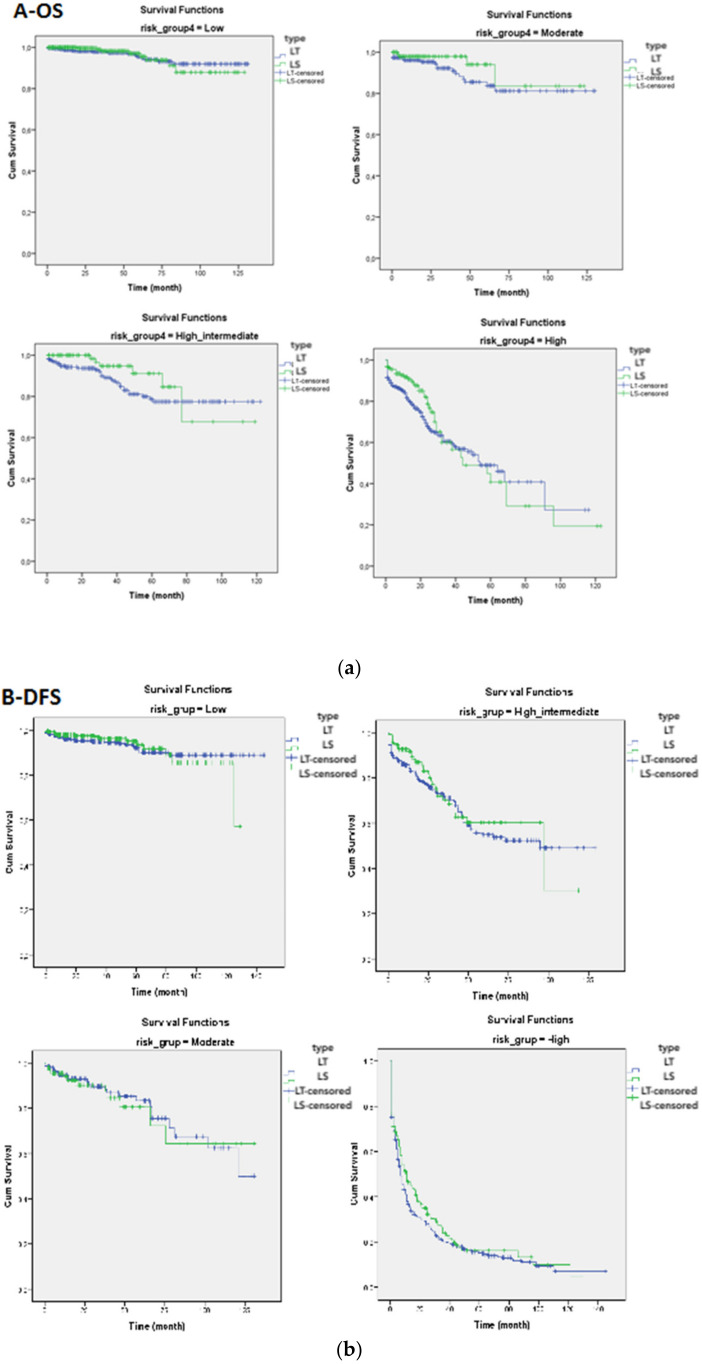
(**a**) Comparison of overall survival (OS) and (**b**) disease-free survival (DFS) curves between the laparotomy and laparoscopy groups according to the low-, moderate-, high–intermediate- and high-risk categories.

The patients were categorized according to the 2016 ESMO-ESGO-ESTRO risk classification system. There were 1226 (46.6%), 363 (13.8%), 389 (14.8%) and 652 (24.8%) patients in the low-, intermediate-, high–intermediate- and high-risk categories, respectively. There was no statistically significant difference between the LS and LT groups according to the subcategories of the 2016 ESMO-ESGO-ESTRO risk classification system in terms of disease-free survival and overall survival (Table 3, Figure 1).

Univariate and multivariate analyses are shown in Table 4 and Table 5, respectively. Only age, BMI, LN status, stage, histopathological type and risk group for disease-free survival and age, BMI, cervical invasion, LN status, and risk group for overall survival, were determined as independent prognostic factors in the multivariate analysis. No superiority between surgical groups was found in the multivariate analysis regarding both disease-free and overall survival rates.

## 4. Discussion

This retrospective multicentric study was conducted to evaluate the oncologic safety and efficacy of conventional laparoscopy in endometrial cancer by stratifying patients with respect to the 2016 ESMO-ESGO-ESTRO risk classification system. Lower pain, lower postoperative complications, shorter hospital stay and recovery and less cost were the well-known advantages of LS compared to LT in numerous studies [2,19]. These short-term advantages are valid for all patients regardless of their risk category. The similar long-term oncological outcomes of LS in low-risk endometrial cancer patients have been demonstrated in the literature to be comparable to open surgery [3,11,13,14,15]. The literature is scarce on the issue of high–intermediate- and high-risk endometrial cancer [18,19,20]. As a reflection of this reality, the European guidelines’ recommendation for the management of low- and intermediate-risk endometrial cancer with minimally invasive surgery (MIS) was level of evidence–I and strength of recommendation–A-, whereas it was considered as level of evidence–IV and strength of recommendation–C- for the management of high-risk endometrial cancer [4]. Consequently, this gap in the literature has inspired us to compare the long-term oncologic outcomes of LS and LT in different risk categories of endometrial cancer. Herein, 2745 endometrial cancer cases were investigated. Of them, 389 (269 LT vs. 120 LS) cases were in the high –intermediate category and 652 (465 LT vs. 187 LS) in the high-risk category. To the best of our knowledge, with the exclusion of the National Cancer Database’s study, on the non-endometrioid uterine cancers including sarcomas by Nieto et al., [24] none of the previous papers compared such a high number of cases. 

As is compatible with the literature, the short-term advantages of LS such as less blood loss, less perioperative and postoperative complications and shorter hospitalization were observed even with the addition of high–intermediate- and high-risk patients in our study. Moreover, the BMI of the LS cohort was significantly higher than the LT group. The mean BMI (38.8 ± 4.7) of our LS group was higher than many published studies, including the landmark study LAP2, which was 28.4 [13,14,18]. In addition, more than half of the LS group had at least one comorbidity. The participating centers in this study are well-known tertiary gynecologic oncology centers in Turkey, which are highly experienced in treating such obese and comorbid patients laparoscopically. Therefore, even with these patients’ characteristics, a sufficient number of LNs were harvested and low conversion rates were obtained. In the LS arm, the resected LN number in patients who underwent pelvic lymphadenectomy was 20.91 and it was 40.93 in pelvic plus para-aortic lymphadenectomy, which is in accordance with the literature [2,14,18,25,26,27,28,29].

The conversion from laparoscopy to laparotomy was required only in 3% of our series, despite the high volume of high-risk patients and even though it is clearly less than those in the landmark LAP2 study (25.8%) and comparable to the following literature, which is mainly below 20% and varies between 0% and 25.8% [2,13,18,19]. The conversion rate in our study was in accordance with the Italian type 2 endometrial cancer series (2.1%) [21]. The low rate of conversion in our series can be attributed to the high expertise of the participating centers of laparoscopic surgery in the gynecological oncology field. According to a multi-centric study in high-volume experienced centers, even when high-risk cases were included, a proposed MIS rate of 80% was found to be an achievable benchmark for the management of women with newly diagnosed endometrial cancer [30]. Hence, patients with endometrial cancer, including high-risk cases, should be referred to centers where this benchmark can be achieved [30].

In our recently published single-institution study, there was no difference in disease-free survival and overall survival between LS and LT groups according to the risk categories [31]. This result is coherent with the previous publications on the comparison of LS and LT in high-risk endometrial cancer [18,19,20,32]. In their multi-centric retrospective study, Fader et al. [18] compared MIS (*n* = 191) and LT (*n* = 192) in type 2 and high-grade endometrial cancer, and they stated that high-risk histopathologic types were not a contraindication for MIS when managed by expert laparoscopists. Fader et al. [32] addressed again in their 2016 publication that patients with grade 3 endometrioid and type 2 endometrial cancer had similar survival outcomes regardless of the surgical approach, MIS or LT. Koskas et al. [19] conducted a comparative study between MIS (*n* = 114) and LT (*n* = 114) in high-risk endometrial cancer. The authors reported identical oncologic outcomes between the groups and concluded that their study provides evidence supporting the use of MIS for high-risk endometrial cancer. Therefore, fear for a poor long-term outcome should not be the reason to refrain from laparoscopic procedures in these patients. [19] Nieto et al. [24] evaluated the impact of MIS on 13,392 patients with stage I-III non-endometrioid uterine cancer (including sarcomas) who underwent hysterectomy between 2010 and 2014 using the National Cancer Database. The authors reported that the route of surgery did not appear to adversely impact survival in these patients [24]. In a multicentric retrospective study by Monterossi et al. [21], 283 patients with type 2 endometrial cancer were evaluated among two groups (LS; 141, LT; 142), and comparable survival outcomes were reported for both of them in stage I–II cases. Favero et al. [20] assessed the oncologic safety of laparoscopic surgery in type 2 endometrial cancer and found that both five-year disease-free and overall survival rates were better among laparoscopically treated patients. However, this superiority was not statistically significant [20]. Consistent with these studies, both the disease-free and overall survival rates of the high–intermediate- and high-risk patients were not statistically different between groups in the current study. However, a possible conflicting impact of race regarding our study population, which consisted only of Turkish women, should be kept in mind. The comparison between the groups, according to the 2016 ESMO-ESGO-ESTRO risk classification system, was a distinguishable characteristic of our study from the former studies. A recently published review on the comparison between MIS and open surgery for high-risk endometrial cancer addressed that MIS showed comparable oncological outcomes and better perioperative and postoperative outcomes than open surgery [22]. However, the authors pointed out the need for prospective randomized studies to approve these results.

Because of the numerous advantages of LS, its importance in endometrial cancer surgery is raising day by day and its increasingly frequent use, more than LT, has been shown in some studies [25,33]. According to the data from the National Cancer Database provided in the study of Nieto and colleagues [24] on non-endometrioid uterine cancers, >50% of hysterectomies were performed with MIS for all of the histologic subtypes (except leiomyosarcoma) by 2014. The authors stated that their findings suggest that MIS has already been widely accepted among clinicians for non-endometrioid uterine cancers, despite the lack of data supporting the procedure’s efficacy and safety. They attributed this condition to the widespread acceptance of MIS for endometrioid endometrial cancers, which likely boosted the acceleration of MIS for other histologic subtypes [24]. However, and even of the limited studies, the availability of comparable oncologic outcomes with MIS versus LT in high-risk endometrial cancer is promising and encouraging. The present study strived to strengthen the assertion that laparoscopy is equal to laparotomy in high–intermediate- and high-risk endometrial cancer patients, in terms of long-term oncologic outcomes. Nevertheless, this result should cautiously be interpreted, considering its retrospective nature and possible selection biases, which is the main weakness of our study. Heterogeneous histologic subtypes, stages and adjuvant treatments were the other limitations. On the other hand, however, pioneering the concept of ESMO-ESGO-ESTRO risk-based comparative survival analysis and including a large number of patients from academic comprehensive cancer centers with expert gynecologic pathologists and gynecologic oncologists who are familiar with LS in gynecologic oncology are the main strengths of our study.

## 5. Conclusions

Regardless of the endometrial cancer risk category, long-term oncologic outcomes of LS are comparable to those treated with LT. Since short-term advantages (fewer complications, short hospitalization and rapid recovery) of LS are also valid for high–intermediate- and high-risk endometrial cancer, it is reasonable to assume that LS has the ability to accelerate treatment with adjuvant therapies in these patients. Therefore, considering the laparoscopic approach as the preferable choice for high–intermediate- and high-risk endometrial cancer as well as for the low- and intermediate-risk categories will probably be the issue of debate for the foreseeable future. Hence, the need for prospective randomized studies on this subject is indisputable.

## Figures and Tables

**Table 1:** Demographic and surgical characteristics of the patients.

Variables		LT		LS		*p*
		(Mean)/*n*	SD/(%)	(Mean)/*n*	SD/(%)	
**Age (years)**		(59,3)	10,7	(57,5)	10,1	<0.05
**Parity**		(3,1)	2,3	(2,7)	1,9	<0.05
**BMI (kg/m^2^**)		(32,1)	5,9	(38,8)	4,7	<0.05
**BMI group**	<30	449	(33,2)	331	(36,8)	<0.001
	30–35	560	(41,5)	258	(28,7)	
	35–40	184	(13,6)	171	(19,0)	
	>40	158	(11,7)	139	(15,5)	
**Comorbidities**	No	565	(34,5)	480	(48,0)	<0.001
	Yes	1072	(65,5)	521	(52,0)	
**Menopausal status**	Premenopausal	307	(35,2)	248	(32,8)	<0.001
	Postmenopausal	566	(64,8)	507	(67,2)	
**Operation type**	TH + BSO	510	(29)	329	(32,9)	<0.001
	TH + BSO + BPLND ± OMENTECTOMY	386	(21,3)	440	(44,0)	
	TH + BSO + BPPALND ± OMENTECTOMY	847	(49,7)	233	(14,2)	
**Perioperative complications**	No	1255	(95,2)	736	(97,0)	<0.001
	Bleeding	54	(4,1)	9	(1,2)	
	Urinary system injury	4	(,3)	8	(1,1)	
	Intestinal injury	2	(,2)	4	(,5)	
	Others	3	(,2)	2	(,3)	
**Postoperative complications**	No	1221	(92,4)	727	(95,7)	<0.001
	Infection	88	(6,7)	15	(2,0)	
	Urinary system injury	0	(,0)	1	(,1)	
	Intestinal injury	1	(,1)	2	(,3)	
	Others	12	(,9)	15	(2,0)	
**Operation time (minute)**		(119,3)	42,8	(142,2)	66,4	<0.05
**Estimated blood loss (mL)**		(243,3)	167,2	(114,6)	81,7	<0.05
**Drop in hemoglobin (gr/dL)**		(1,6)	1,1	(1,4)	1,1	<0.05
**Conversion of laparoscopy to laparotomy**				27	(3,0)	
**Postoperative hospitalization (day)**		(5.4)	3.3	(3.3)	1.7	<0.05

LT: laparotomy, LS: laparoscopy, *n*: count, SD: Standard deviation, BMI: body mass index, TH + BSO: total hysterectomy bilateral salpingo-oophorectomy, BPLND: bilateral pelvic lymph node dissection, BPPALND: bilateral pelvic para-aortic lymph node dissection.

**Table 2:** Histopathological features of the groups.

Variables		LT		LS		*p*
		(Mean)/*n*	SD/(%)	(Mean)/*n*	SD/(%)	
**Stage**	1a	825	(47,3)	664	(66,2)	<0.001
	1b	372	(21,4)	195	(19,4)	
	2	130	(7,4)	31	(3,1)	
	3a + 3b + 3c + 4	414	(23,9)	114	(11,3)	
**Histopathology**	Endometrioid	1280	(79,1)	875	(87,9)	<0.001
	Serous	113	(7,0)	38	(3,8)	
	Clear	33	(2,0)	2	(,2)	
	Mixed	128	(7,9)	67	(6,7)	
	Carcinosarcoma	65	(4,0)	14	(1,4)	
**Histologic type**	Type1	1280	(79,1)	875	(87,9)	<0.001
	Type2	339	(20,9)	121	(12,1)	
**Grade**	1	715	(43,9)	537	(54,7)	<0.001
	2	524	(32,2)	342	(34,9)	
	3	389	(23,9)	102	(10,4)	
**Myometrial invasion**	No	324	(20,1)	239	(24,0)	<0.001
	<%50	670	(41,6)	506	(50,8)	
	≥%50	615	(38,2)	252	(25,3)	
**Cervical invasion**	No	1065	(83,5)	711	(95,4)	<0.001
	Yes	211	(16,5)	34	(4,6)	
**LVSI**	No	988	(64,0)	749	(77,3)	<0.001
	Yes	555	(36,0)	220	(22,7)	
**LN count**	No lymphadenectomy	510	(29,3)	329	(32,7)	0.037
	1–20	460	(26,3)	267	(26,7)	
	21–40	453	(26,1)	259	(25,9)	
	>40	320	(18,3)	147	(14,6)	
**Metastatic LN**	No	1512	(86,8)	931	(93,0)	<0.001
	Pelvic	103	(5,9)	46	(4,6)	
	Paraaortic	41	(2,3)	8	(0,7)	
	Pelvic + paraaortic	87	(5,0)	17	(1,7)	

LT: laparotomy, LS: laparoscopy, *n*: count, SD: Standard deviation, LVSI: lymphovascular space invasion; LN: lymph node.

**Table 3:** Comparison of DFS and OS rates between groups with respect to the ESMO-ESGO-ESTRO risk classification system.

Risk Group	DFS					OS				
	LT		LS			LT		LS		
	Total *n*/Censored%	Mean	Total *n*/Censored%	Mean	*p*	Total *n*/Censored%	Mean	Total *n*/Censored%	Mean	*p*
**Low**	668/96,4	121,6	564/98,2	120,7	0,274	668/96,9	122,8	564/98,4	121,2	0,320
**Intermediate**	234/84,2	89,6	130/91,5	105,3	0,180	234/90,6	109,7	130/96,2	110,8	0,129
**High-intermediate**	271/75,6	80,0	121/75,2	77,7	0,366	271/87,8	101,9	121/94,2	98,2	0,153
**High**	464/53,2	32,4	186/54,3	23,9	0,106	464/72,8	60,5	186/78,0	58,8	0,231
***p***		<0.001		<0.001			<0.001		<0.001	

DFS: Disease-free survival; OS: Overall survival; LT: laparotomy, LS: laparoscopy, *n*: count.

**Table 4:** Univariate analysis of DFS and OS.

Variables	Total/Dead *n*	DFS Mean	OS Mean
**Metastatic lymph node**			
No	2317/132	98.5	115.9
Yes	283/109	19.5	44.9
*p*		<0.001	<0.001
**Stage**			
1a	1502/47	120.0	121.3
1b	567/40	74.0	112.5
2	162/31	52.8	82.5
3a + 3b + 3c + 4	394/130	23.4	54.9
*p*		<0.001	<0.001
**Grade**			
1	1208/49	122.1	129.3
2	798/63	92.3	114.1
3	231/51	24.0	74.6
*p*		<0.001	<0.001
**Myometrial invasion**			
None	867/153	119.3	121.3
<%50	1176/64	110.4	115.2
≥%50	271/80	51.5	93.3
*p*		<0.001	<0.001
**Lymphovascular space invasion**			
No	1737/77	110.9	118.4
Yes	775/154	51.8	85.3
*p*		<0.001	<0.001
**Histopathological type**			
Type 1	2155/141	103.9	114.7
Type 2	460/103	30.9	62.2
*p*		<0.001	<0.001

DFS: Disease-free survival, OS: Overall survival, *n*: count.

**Table 5:** Multivariate analysis of DFS and OS.

Variables	HR (95,0% CI)			
	DFS	*p*	OS	*p*
Age	**1,025 (1,012–1,038)**	**<0.001**	**1,038 (1,020–1,055)**	**<0.001**
Surgery type	1,055 (0,869–1,282)	0.587	1,338 (0,895–1,999)	0.156
Comorbidities	0,980 (0,762–1,262)	0.877	0,959 (0,669–1,374)	0.820
BMI <30	ref			
30–40	1,281 (0,946–1,735)	0.109	**1,831 (1,150–2,914)**	**0.011**
>40	**1,386 (1,032–1,863)**	**0.003**	**2,133 (1,353–3,361)**	**0.001**
MI	ref			
None	0,689 (0,434–1,095)	0.115	0,640 (0,381–1,076)	0.092
<%50	1,008 (0,599–1,698)	0.975	0,770 (0,427–1,387)	0.383
>%50	0,887 (0,618–1,274)	0.517	0,713 (0,398–1,275)	0.253
LVSI	1,374 (0,987–1,912)	0.060	**1,982 (1,288–3,050)**	**0.002**
Cervical invasion	**1,713 (1,131–2,595)**	**0.011**	**3,139 (1,662–5,926)**	**<0.001**
Metastatic LN	ref			
Stage 1a	1,051 (0,669–1,652)	0.828	0,576 (0,268–1,241)	0.159
1b	**1,847 (1,083–3,150**)	**0.024**	1,379 (0,623–3,052)	0.427
2	**2,012 (1,226–3,303)**	**0.006**	1,519 (0,685–3,368)	0.304
3a + 3b + 3c + 4	ref			
Grade 1	0,428 (0,313–0,585)	0.146	0,643 (0,392–1,054)	0.280
2	0,827 (0,641–1,067)	0.761	0,791 (0,518–1,210)	0.458
3	**1,973 (1,495–2,605)**	**<0.001**	1,462 (0,974–2,196)	0.067
Histopathological type	ref			
Risk group Low	1,204 (0,935–1,549)	0.082	**2,411 (1,076–5,405)**	**0.036**
Intermediate	**2,833 (1,452–5,529)**	**0.002**	**2,406 (1,084–5,337)**	**0.019**
High–intermediate	**6,349 (3,569–11,294)**	**<0.001**	**3,216 (1,482–6,978)**	**<0.001**
High				

HR: Hazard Ratio; CI: Confidence Interval; DFS: Disease-free survival; OS: Overall survival; BMI: Body mass index; MI: Myometrial invasion; LVSI: Lymphovascular space invasion; LN: Lymph node.

## Data Availability

Data are available to be shared with authorities only upon a reasonable request due to the patients’ privacy and ethics rules issues.
